# Standardization of T1w/T2w Ratio Improves Detection of Tissue Damage in Multiple Sclerosis

**DOI:** 10.3389/fneur.2019.00334

**Published:** 2019-04-09

**Authors:** Graham Cooper, Carsten Finke, Claudia Chien, Alexander U. Brandt, Susanna Asseyer, Klemens Ruprecht, Judith Bellmann-Strobl, Friedemann Paul, Michael Scheel

**Affiliations:** ^1^NeuroCure Clinical Research Center, Charité – Universitätsmedizin Berlin, Corporate Member of Freie Universität Berlin, Humboldt-Universität zu Berlin and Berlin Institute of Health, Berlin, Germany; ^2^Einstein Center for Neurosciences, Berlin, Germany; ^3^Experimental and Clinical Research Center, Max Delbrueck Center for Molecular Medicine and Charité – Universitätsmedizin Berlin, Corporate Member of Freie Universität Berlin, Humboldt-Universität zu Berlin and Berlin Institute of Health, Berlin, Germany; ^4^Berlin School of Mind and Brain, Humboldt-Universität zu Berlin, Berlin, Germany; ^5^Department of Neurology, University of California, Irvine, Irvine, CA, United States; ^6^Department of Neurology, Charité – Universitätsmedizin Berlin, Corporate Member of Freie Universität Berlin, Humboldt-Universität zu Berlin and Berlin Institute of Health, Berlin, Germany; ^7^Department of Neuroradiology, Charité – Universitätsmedizin Berlin, Corporate Member of Freie Universität Berlin, Humboldt-Universität zu Berlin and Berlin Institute of Health, Berlin, Germany

**Keywords:** magnetic resonance imaging techniques, multiple sclerosis, normal appearing white matter, T1w/T2w ratio, relapsing-remitting

## Abstract

Normal appearing white matter (NAWM) damage develops early in multiple sclerosis (MS) and continues in the absence of new lesions. The ratio of T1w and T2w (T1w/T2w ratio), a measure of white matter integrity, has previously shown reduced intensity values in MS NAWM. We evaluate the validity of a standardized T1w/T2w ratio (sT1w/T2w ratio) in MS and whether this method is sensitive in detecting MS-related differences in NAWM. T1w and T2w scans were acquired at 3 Tesla in 47 patients with relapsing-remitting MS and 47 matched controls (HC). T1w/T2w and sT1w/T2w ratios were then calculated. We compared between-group variability between T1w/T2w and sT1w/T2w ratio in HC and MS and assessed for group differences. We also evaluated the relationship between the T1w/T2w and sT1w/T2w ratios and clinically relevant variables. Compared to the classic T1w/T2w ratio, the between-subject variability in sT1w/T2w ratio showed a significant reduction in MS patients (*p* < 0.001) and HC (*p* < 0.001). However, only sT1w/T2w ratio values were reduced in patients compared to HC (*p* < 0.001). The sT1w/T2w ratio intensity values were significantly influenced by age, T2 lesion volume and group status (MS vs. HC) (adjusted R^2^ = 0.30, *p* < 0.001). We demonstrate the validity of the sT1w/T2w ratio in MS and that it is more sensitive to MS-related differences in NAWM compared to T1w/T2w ratio. The sT1w/T2w ratio shows promise as an easily-implemented measure of NAWM in MS using readily available scans and simple post-processing methods.

## Introduction

Multiple sclerosis (MS) is an inflammatory and neurodegenerative CNS disease characterized by focal lesions with demyelination, axonal loss, and reactive gliosis in white matter (WM) and gray matter (GM) (1–5). MRI plays a critical role in the diagnosis and monitoring of MS patients through detection of lesions in WM and GM (6). Although tissue damage in the WM beyond lesions is frequently found in neuropathological examinations of MS, it is not detectable in routine clinical MRI scans. This has led to the term normal-appearing WM (NAWM) for WM that appears normal on routine MRI but may contain neuropathologically detectable tissue damage. NAWM damage can be demonstrated using advanced MRI techniques such as diffusion tensor imaging (7–9) or other quantitative approaches (10). Using these approaches, it has been shown that NAWM alone can discriminate MS patients from healthy subjects (10), indicating the high prevalence and sensitivity of NAWM in MS. A clear draw-back of such techniques is the need for time-intensive additional scans and expertise in image post-processing. Glasser and Van Essen proposed that the ratio of T1w and T2w images (T1w/T2w ratio) in the GM can be used to create an estimate of cortical myelin content in the healthy population (11–13). The T1w/T2w ratio has also been applied to the whole brain and WM (14, 15). Although the sensitivity of the T1w/T2w ratio to myelin has been contested, there is growing consensus that the T1w/T2w ratio is a marker of general WM microstructure (15–17). Recently, reduced T1w/T2w ratio intensity values have been found in the cortex and NAWM of MS patients compared to HC (15, 17).

However, the T1w/T2w ratio has technical limitations. It is long established that qualitative T1w and T2w intensity values are variable and depend on numerous technical and methodological factors, such as field strength and scanner manufacturer, leading to problems in comparing these values (and quantitative parameters derived from these, such as volume) between different scanners and time-points (18–21). This may limit a reliable and valid comparison of T1w/T2w ratio intensity values across subjects. Recently, Misaki et al. showed that standardizing the T1w/T2w ratio can lead to enhanced delineation of tissue classes (22). They standardize the T1w/T2w based on the median GM intensity in the T1w and T2w images and produce intensity values in the range of−1 to 1, where WM intensity values are positive, cerebrospinal fluid (CSF) intensity values are negative and GM intensity values are between −0.01 and 0.01. This standardized T1w/T2w ratio (sT1w/T2w ratio) reduces the between-subject variability of intensity values and may overcome the described limitation of the T1w/T2w ratio therefore allow a more valid comparison of intensity values in NAWM.

The current study aimed to evaluate the validity of the standardization of the T1w/T2w ratio in MS patients. We hypothesized that (1) the sT1w/T2w ratio would significantly reduce the between subject variability in NAWM in MS patients compared to the T1w/T2w ratio, and (2) that NAWM group differences between healthy controls (HC) and MS patients would be more pronounced using the sT1w/T2w ratio. In addition, we evaluated clinically relevant covariates of sT1w/T2w.

## Materials and Methods

### Patients and Controls

Patient and HC data were taken from an observational study, approved by the institutional review board (EA1/163/12, EA1/189/13). HC matching patients for sex and age (+/−6 months) were identified using in-house python scripts using Python 3. Overall, 47 patients and 47 HC were included in the study (Table 1).

**Table 1:** Demographic and clinical characteristics.

	**RRMS**	**HC**
*N*	47	47
Sex	30 female	30 female
Mean age (yrs) (sd) (range)	37.69 (9.22) (21.5–60.2)	36.23 (9.73) (20.5–63.7)
Median EDSS (range)	2.00 (0.00–6.00)	NA
Mean disease duration (months) (sd)	24.6 (57.57)	NA
Mean T2 lesion volume (ml) (sd)	5.07 (6.86)	0.07 (0.09)
Mean T2 lesion count (sd)	59.23 (46.75)	3.09 (3.58)

All patients and HC provided written informed consent. MS patients had a diagnosis of relapsing remitting MS (RRMS) according to 2010 McDonald criteria (23). Clinical assessment was performed by experienced neurologists and included the Expanded Disability Status Scale (EDSS) (24).

### MRI Acquisition

MRI acquisition was performed on a 3 Tesla MRI (Tim Trio, Siemens Medical Systems, Erlangen, Germany). The MRI protocol included a 3D MPRAGE (T1w, TR = 1,900 ms, TE = 2.55 ms, TI = 900 ms, 1 mm isotropic resolution), a 3D T2SPACE (T2w, TR = 5,000 ms, TE = 502 ms, 1 mm isotropic resolution) sequence and a 3D FLAIR (TR = 6,000 ms, TE = 388 ms, TI = 2,100 ms, 1 mm isotropic resolution).

### MRI Analysis

Before calculation of the T1w/T2w and sT1w/T2w ratio, the T1w, T2w and FLAIR images were preprocessed as follows: All images were bias field corrected using non-parametric non-uniform intensity normalization (25), changed to a robust field of view and oriented to MNI space using FSL tools. The T2w and FLAIR images were then co-registered to the corrected T1w image and these three images were then registered to standard MNI space. Co-registration and registration to standard space was performed using a spline interpolation with FSL FLIRT (26, 27). Lesion segmentation was done semi-automatically on FLAIR using the lesion prediction algorithm [LPA (28)] as implemented in the Lesion Segmentation Toolbox version 2.0.15 (www.statistical-modelling.de/lst.html). Lesion masks were subsequently manually corrected using ITK-SNAP (29) (www.itksnap.org). Generation of a brain mask and tissue segmentation into GM, WM, and cerebrospinal fluid (CSF) was achieved using the Computational Anatomy Toolbox version 11.09 (30) implemented in SPM12 version 7219. Each lesion mask was subtracted from the WM and GM masks, to create a normal-appearing GM (NAGM) and NAWM mask, respectively. Median intensity values in the T1w and T2w were extracted in NAGM, NAWM, and CSF for each subject.

### T1w/T2w Ratio and sT1w/T2w Ratio Calculation

The T1w/T2w ratio was calculated using the FSL stats tool (31) by dividing the processed 3D T1w image by the spatially registered 3D T2w image.

In order to calculate the sT1w/T2w ratio, a scaling factor was found by dividing the median NAGM intensity value in the T1w image by the median NAGM intensity value in the T2w image. The T2w image was then multiplied by the scaling factor to create a scaled T2w image (sT2). The sT1w/T2w ratio was then calculated using the equation from Misaki et al. (22):

$$\begin{array}{l} \frac{sT1w}{T2w}ratio=\;\frac{T1w-sT2}{T1w+sT2} \end{array}$$

Finally, median T1w/T2w and sT1w/T2w values were extracted from the NAGM, NAWM, and CSF for each subject.

### Statistical Analysis

Statistical analysis was conducted in R (32). *t*-tests and χ^2^ tests were used to assess group differences in age and sex, respectively. All figures shown were created using the tidy verse and ggpubr (33, 34).

First, the between-subject variation in NAWM in T1w/T2w and sT1w/T2w was measured using the coefficient of variation (CoV) and compared using the Feltz & Miller test with the cvequality package (35). We then compared group differences of each measure (T1w, T2w, T1w/T2w, sT1w/T2w) between MS patients and HC in NAGM and NAWM intensity using *t*-tests. The relationship between T1w/T2w and sT1w/T2w ratio intensity values was also investigated using Pearson's correlation.

In order to evaluate relevant covariates of each measure in NAWM, we analyzed the median intensity values of T1w, T2w, T1w/T2w, and sT1w/T2w using linear models with a backwards, stepwise method: A model was built for each tissue intensity, with group, age, and sex included as predictors. This model was then reduced, removing one non-significant predictor at a time until the model contained only significant predictors. Model fit was assessed using the Akaike Information Criteria (36), where lower values signify better fit, and the best-fitting model was selected. Following the recommendations of Rippon et al. (37), sex was not included on its own, but rather as an interaction term with head size [V-scaling factor from SIENAX (38)].

Finally, we investigated the clinically relevant covariates of measures in NAWM of patients using the same method to build linear models. Age, the interaction between sex and head size, T2 lesion volume and count, EDSS, and disease duration were included as predictors in the initial model. The interaction between disease duration and age was also included. The stepwise linear regression for both analyses was conducted using the Modern Applied Statistics with S package (39).

Each linear regression was also tested for effects of extreme values by assessing Cook's difference. Cook's difference of >1 would indicate that an extreme value was overly influencing the regression model. The significance threshold for all analyses was set at *p* < 0.004, using Bonferroni correction for multiple comparisons.

## Results

### Demographics

MS patients and HC were well-matched in regard to sex (χ^2^ = 0.00 [1.00] *p* = 1.000) and age (*t* = −0.75, [91.74] *p* = 0.456), shown in Table 1.

### Intensity Value Distribution

**Figure 1:**
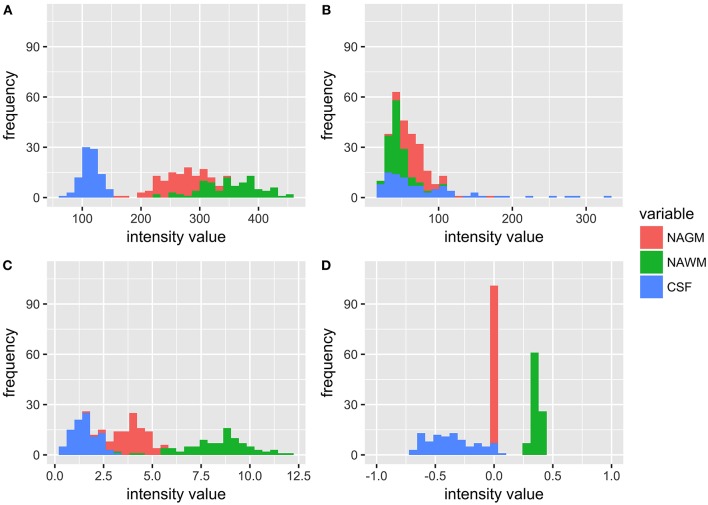
Histograms showing the distribution of intensity values for all patients and healthy controls in NAGM, NAWM, and CSF in **(A)** T1w, **(B)** T2w, **(C)** T1w/T2w, and **(D)** sT1w/T2w images. It can be seen that the T1w/T2w ratio reduces overlap in tissue intensity values but not to the same extent as sT1w/T2w, where all NAGM values are around 0, all NAWM values range from 0 to 1, and all CSF values range from −1 to 0.

**Figure 2:**
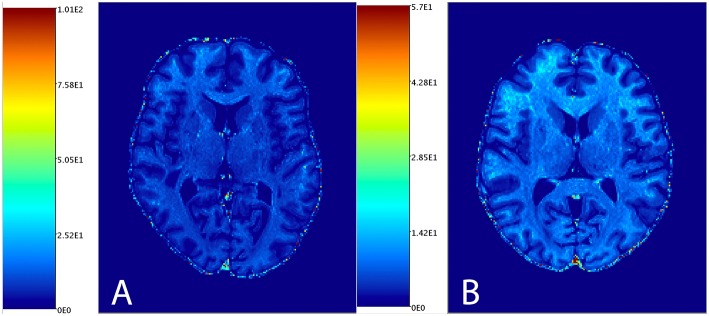
An example T1w/T2w image from one patient **(A)** and one healthy subject **(B)**.

**Figure 3:**
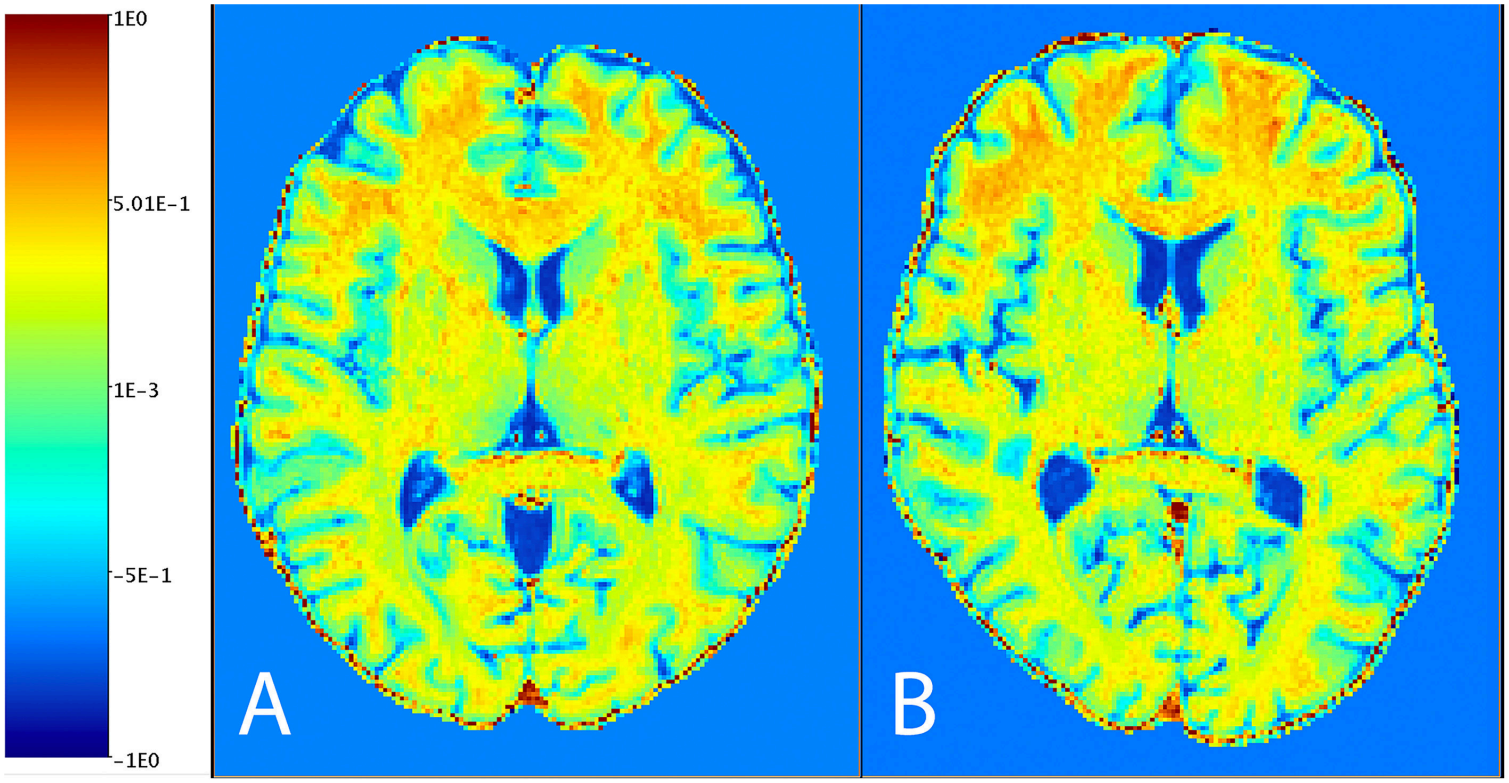
An example sT1w/T2w image from one patient **(A)** and one healthy subject **(B)**.

Figure 1 shows the distribution of NAGM, NAWM and CSF intensity values in each of the four image types (T1w, T2w, T1w/T2w, and sT1w/T2w). An example T1w/T2w and sT1w/T2w ratio intensity map for two patients and two healthy controls is shown in Figures 2, 3. The CoV of NAWM intensity values was significantly reduced in sT1w/T2w compared to T1w/T2w in both patients and controls (Table 2). As expected, T1w/T2w and sT1w/T2w ratio intensity values were found to significantly correlate (adjusted R^2^ = 0.23).

**Table 2:** Comparison of NAWM intensity value CoV in T1w/T2w and sT1w/T2w.

	**T1w/T2w**	**sT1w/T2w**	**Significance**
MS	22.04	9.82	*p <* 0.001
HC	17.02	7.06	*p <* 0.001

### Group Analysis

**Figure 4:**
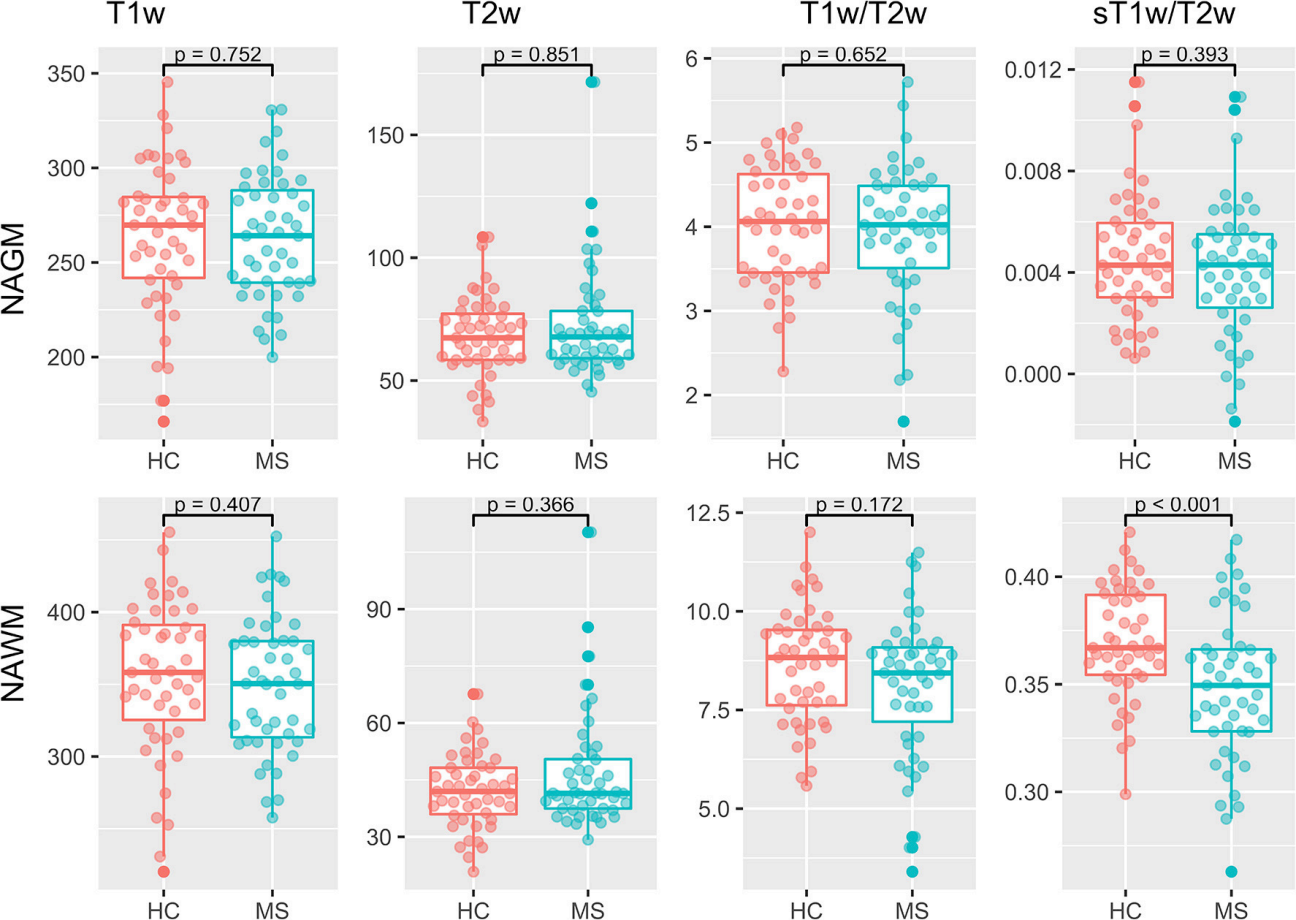
Group comparisons for the median NAGM and NAWM intensity values in T1w, T2w, T1w/T2w, and sT1w/T2w images. No significant differences were found between MS and HC in any image except sT1w/T2w using a *t*-test.

Figure 4 shows the results of the group comparisons of each tissue type in each of the T1w, T2w, T1w/T2w, and sT1w/T2w images. Differences between MS patients and HC were only found in sT1w/T2w ratio intensity values of the NAWM. Table 3 shows the results of the original and (where applicable) best-fitting regression models for NAWM intensity values in T1w, T2w, T1w/T2w, and sT1w/T2w images. Variation in neither T1w, T2w nor T1w/T2w NAWM intensity values showed significant associations to age, sex, or group (MS/HC) surviving multiple comparison correction. MS diagnosis and age explained variation in sT1w/T2w ratio NAWM intensity values (adjusted R^2^ = 0.23). No model contained extreme values according to the Cook's distance criteria.

**Table 3:** Regression equation results for the NAWM intensity in each image (T1w, T2w, T1w/T2w ratio, and sT1w/T2w ratio).

	**Dependent variable**				
	**T1w**	**T2w**	**T1w/T2w**	**sT1w/T2w**	
	**Original**	**Original**	**Original**	**Original**	**Final**
MS diagnosis	−1.34 (0.895)	4.36 (0.093)	−0.37 (0.256)	**−0.02 (0.003)**	**−0.02 (0.001)**
Age	−1.04 (0.059)	−0.28 (0.044)	0.01 (0.469)	**−0.001 (< 0.001)**	**−0.001 (< 0.001)**
Female^*^	−23.48 (0.620)	8.73 (0.466)	−3.68 (0.017)	0.002 (0.945)	
Male^*^	−45.14 (0.391)	9.74 (0.464)	−4.04 (0.019)	−0.003 (0.926)	
Constant	**432.94 (< 0.0001)**	40.14 (0.020)	**13.22 (< 0.0001)**	**0.415 (< 0.0001)**	**0.41 (< 0.0001)**
Observations	93	93	93	93	94
Adjusted R^2^	0.06	0.03	0.04	0.22	0.21
Residual SE	48.48	12.26	1.56	0.03	0.03
F statistic (df)	2.38 (4, 88)	1.76 (4, 88)	1.98 (4, 88)	7.34 (4, 88)	13.06 (2, 91)
AIC	992.66	737.02	353.28	−394.12	−396.40

*The original model with all variables is presented for each image. If a significant model was found as a result of the backwards stepwise regression with better fit (according to the AIC criterion), this final model is also presented. The standardized coefficient is shown for each parameter with the p-value in brackets. Parameters significant after correction for multiple comparisons are highlighted in bold*.

* *interaction with head size*.

### Clinically Relevant Covariates of NAWM

Table 4 shows the results of the original and (where applicable) best-fitting regression models for NAWM intensity values in T1w, T2w, T1w/T2w, and sT1w/T2w images. Patients' sT1w/T2w ratio NAWM intensity values were explained by T2 lesion count (adjusted R^2^ = 0.47). No variation in the patient NAWM T1w, T2w, or T1w/T2w ratio was significantly accounted for by any parameters after correcting for multiple comparisons. No model contained extreme values according to the Cook's distance criteria.

**Table 4:** Regression equation results for patients' NAWM intensity in each image (T1w, T2w, T1w/T2w ratio, and sT1w/T2w ratio).

	**Dependent variable**				
	**T1w**	**T2w**	**T1w/T2w**	**sT1w/T2w**	
	**Original**	**Original**	**Original**	**Original**	**Final**
Age	−0.163 (0.893)	−0.279 (0.3232)	0.006 (0.8776)	−0.001 (0.07972)	−0.001 (0.0121)
Disease duration	0.414 (0.634)	0.138 (0.4938)	−0.021 (0.4776)	0.0001 (0.89587)	
EDSS	−6.356 (0.291)	−1.142 (0.4104)	0.072 (0.7240)	−0.002 (0.48957)	
T2 lesion volume	0.0004 (0.745)	0.001 (0.0308)	0.00003 (0.4559)	0 (0.53510)	
T2 lesion count	−0.183 (0.345)	−0.050 (0.2684)	−0.0005 (0.9436)	**−0.0004 (0.00142)**	**−0.0004 (< 0.0001)**
Age^*^	−0.013 (0.472)	−0.001 (0.7258)	0.0002 (0.6856)	0 (0.87527)	
Female^**^	−74.163 (0.247)	1.690 (0.9083)	−5.654 (0.0127)	−0.012 (0.75983)	
Male^**^	−99.270 (0.157)	2.621 (0.8696)	−6.592 (0.0079)	−0.018 (0.65231)	
Constant	**498.225 (< 0.0001)**	49.772 (0.0300)	**16.432 (< 0.0001)**	**0.448 (< 0.0001)**	**0.415 (< 0.0001)**
Observations	45	45	45	45	47
Adjusted R^2^	0.130	0.163	0.085	0.419	0.361
Residual SE	43.828	10.124	14.500	0.026	0.027
F statistic (df)	1.821 (8, 36)	2.068 (8, 36)	5.287 (8, 36)	4.961 (8, 36)	13.992 (2, 44)
AIC	477.8878	388.7079	173.9892	−190.8811	−200.2014

*The original model with all variables is presented for each image. If a significant model was found as a result of the backwards stepwise regression with better fit (according to the AIC criterion), this final model is also presented. The standardized coefficient is shown for each parameter with the p-value in brackets. Parameters significant after correction for multiple comparisons are highlighted in bold*.

* *interaction with disease duration*,

** *interaction with head size*.

## Discussion

Our study evaluated the validity of the standardization of the T1w/T2w ratio in MS. We showed that (1) CoV of NAWM was reduced in the sT1w/T2w ratio compared to the T1w/T2w ratio, and (2) that NAWM group differences were more pronounced between HC and MS using the sT1w/T2w ratio. We also show the clinically relevant covariates of NAWM T1w/T2w and sT1w/T2w ratio intensity values. Group (MS vs. HC), age and T2 lesion volume explained variation in the sT1w/T2w ratio intensity values, whereas the T1w/T2w ratio was only sensitive to sex.

### Benefits of the sT1w/T2w Ratio

The sT1w/T2w ratio method proposed by Misaki et al. (22) uses a scaling factor derived from median GM intensity values in T1w and T2w images to standardize the T1w/T2w ratio. This method improves the T1w/T2w ratio by creating scaled intensity values: GM intensity values are scaled to 0, WM values are scaled between 0 and 1, and CSF values are scaled between 0 and −1. We propose that this scaling results in values that can be more meaningfully compared between subjects, scanners and time-points. As discussed above, the T1w/T2 ratio produces variable intensity values that may be affected by a number of technical and methodological parameters, such as field strength and scanner manufacturer. Scaling the T1w/T2w ratio intensity values according to a non-varying value should overcome these limitations, increasing the reliability of the method and presenting a more informative bio-marker.

In the context of MS, where lesions or subtle changes within the GM could alter T1w and T2w intensity values and could influence the scaling, it is necessary to control for these factors. Therefore, median values were extracted from GM not affected by lesions, i.e., NAGM. In addition, we found that T1w and T2w NAGM values do not significantly differ between MS patients and HC. The standardization method was also shown to significantly reduce the CoV in NAWM in both HC and MS patients. As such, the sT1w/T2w ratio is a promising measure of NAWM in MS patients, which can be meaningfully compared between MS patients and HC. It is important to note that the standardization of the T1w/T2w ratio also acts as a bias field correction, reducing the heterogeneity of mean intensity values (22). To allow a fair comparison of the T1w/T2w-ratios against the T1w and T2w images, a bias field correction of the T1w and T2w images is included in our preprocessing.

### Normal Appearing White Matter in MS

NAWM is a relevant and important aspect of pathology that has been demonstrated to influence cognitive impairment in MS (40, 41). Although the T1w/T2w ratio has been criticized for a lack of specificity to myelin, it should be emphasized that NAWM pathology is not limited to myelin damage. Similar to lesional WM, NAWM is subject to axonal injury, demyelination, inflammation, and gliosis (42–44). Damage in NAWM is widespread and ongoing microstructural changes were shown even in RRMS patients who showed no evidence of disease activity (no new lesions and no increased EDSS score) at follow-up (45). Myelin water loss of up to 8%, indicating reduced myelin in NAWM, has been demonstrated in a 5-years longitudinal study of RRMS patients (46). Reflecting the non-myelin-specific nature of NAWM damage, axonal injury in NAWM has also been demonstrated in RRMS patients using kurtosis imaging (47). It is currently unclear whether NAWM damage is a direct result of lesions, a general neurodegenerative process in the WM, or both (44, 46). NAWM damage has been detected from the onset of disease and prior to lesions (48), which would indicate a general degenerative process. On the other hand, NAWM damage has been shown to correlate with lesions in T2w images (49). Here, we show that NAWM damage, as measured by the sT1w/T2w ratio, is correlated with lesions in T2w images.

In relation to NAWM evaluation in MS, the major finding of the current study is that the sT1w/T2w, and not T1w/T2w ratio intensity values, are significantly reduced in the NAWM of MS patients compared to HC. Previous work has shown significant reduction in NAWM T1w/T2w ratio intensity values (15). There are a number of differences between the study by Beer et al. and the current study. One explanation for the difference in findings may be or the decision to use median values in the current study [in comparison to mean values, which are more susceptible to effects of outliers (50)]. Beer et al. had a larger sample size (244 MS patients and 78 healthy subjects), suggesting they may have had more power to find an effect. An alternative explanation for the disparity in findings relates to the discussed variability in T1w and T2w ratio intensity values. We propose that, in addition to the demonstrated enhanced sensitivity of the sT1w/T2w ratio, the standardization of intensity values also increases the reliability of comparisons between groups, overcoming the variability of raw intensity values. This should be investigated in future work using different scanners or longitudinal data.

While the finding of differences in NAWM in MS patients is not new, the majority of previous studies reporting this have used advanced MRI techniques, such as myelin water imaging approaches (e.g., multi-echo T2 mapping) and diffusion-based imaging approaches (diffusion tensor imaging (DTI) and diffusion kurtosis imaging) (51, 52). DTI measures microstructural integrity and has been used to demonstrate abnormalities in NAWM tracts in MS (45, 53). Similarly, the myelin water fraction has been shown to be decreased in MS patients compared to HC (46, 54). The myelin fraction is proposed to be the most sensitive MRI measure of myelin to date (55). A clear drawback of the myelin water fraction and DTI is that they are not commonly acquired as part of the standard clinical MRI protocol and they require expertise in image post-processing. We show similar results using the sT1w/T2w ratio, which requires only T1w and T2w scans and a very simple post-processing procedure. As such, this technique can be readily incorporated into the clinical routine and extends the amount of clinically-relevant information that can be obtained from these scans.

### Limitations of the sT1w/T2w Ratio

The standardization approach normalizes intensity values using NAGM intensity values as a reference (scaling factor). This reduces NAGM values to 0, which results in poor applicability of the sT1w/T2w ratio in the NAGM. However, as discussed above, the use of NAGM as a scaling factor also ensures that sT1w/T2w ratios are comparable between patients and HC, as previous work has shown detectable cortical pathology in GM (17).

One important limitation of the T1w/T2w ratio that also limits the sT1w/T2w ratio is the non-specificity of this marker and the lack of a clear biological/pathological substrate for changes in values. T1w and T2w intensity values (and, by extension, T1w/T2w and sT1w/T2w ratio values) are affected by a range of microstructural components such as paramagnetic hemoglobin, iron accumulation and tissue calcification (56), not simply myelin and axonal content. As such, the underlying theory of the T1w/T2w ratio proposed by Glasser and van Essen (12), may be too simple and does not necessarily reflect myelin specifically. Evidence for the non-specificity of the T1w/T2w ratio to myelin also comes from imaging studies; the myelin water fraction, which has been validated as a specific marker of myelin (46, 57), has been shown to have no correlation with T1w/T2w ratio intensity values in WM of healthy subjects (54). Further, pathological investigations of T1w/T2w ratio intensity values show mixed results and have focused on cortical myelination. Glasser and van Essen demonstrated that the T1w/T2w ratio is visually comparable to myeloarchitectonic maps of the cortex, suggesting a relationship with myelin (11). In support of Glasser and van Essen, Nakamura et al. compared myelinated and non-myelinated tissue of MS patients and found a significant reduction of T1w/T2w values in non-myelinated tissue (58).

However, as discussed above, myelinated and non-myelinated tissue in MS are not exclusively based on myelin levels and hence, it cannot be directly interpreted as a marker of myelination. Rather, it has been posited that the T1w/T2w ratio is a general measure of WM microstructure (16, 54, 59), which may be more affected by axonal diameter than myelin density(60). Supporting this, Righart et al. investigated cortical T1w/T2w ratio values in secondary progressive MS brains and found that T1w/T2w intensity values were correlated with dendrite density, not myelin (17). As described above, NAWM pathology in MS is not limited to demyelinating processes and axonal loss has been emphasized as an important process in NAWM (44). Hence, we advise against interpreting sT1w/T2w intensity values as a marker of myelin and rather suggest that the sT1w/T2w ratio intensity values are a measure of general NAWM microstructural integrity.

## Conclusion

We have demonstrated the validity of standardizing the T1w/T2w ratio in an MS cohort. The sT1w/T2w ratio reduces CoV in NAWM of both MS patients and HC and was the only investigated measure to detect differences in NAWM in MS compared to HC. We propose that the sT1w/T2w ratio is a reliable and sensitive measure that can be used to investigate NAWM changes in MS.

## Author Contributions

GC, MS, CF, and FP contributed to the conception and design of the study. MS, JB-S, KR, and SA were involved in the acquisition and organization of clinical and/or MRI data. GC performed the statistical analysis and wrote the first draft of the manuscript. MS, AB, and CC verified the analytical methods. MS, FP, CF, and AB supervised the finalization of the manuscript. All authors contributed to manuscript revision and read and approved the submitted version.

### Conflict of Interest Statement

KR was supported by the German Ministry of Education and Research (BMBF/KKNMS, Competence Network Multiple Sclerosis) and has received research support from Novartis and Merck Serono as well as speaking fees and travel grants from Guthy Jackson Charitable Foundation, Bayer Healthcare, Biogen Idec, Merck Serono, sanofi-aventis/Genzyme, Teva Pharmaceuticals, Roche and Novartis. AB is cofounder and shareholder of technology start-ups Motognosis and Nocturne. He is named as inventor on several patent applications describing MS serum biomarkers, perceptive visual computing for motor function assessment and retinalimage analysis. JB-S has received travel grants and speaking fees from Bayer Healthcare, Biogen Idec, Merck Serono, sanofi-aventis/Genzyme, Teva Pharmaceuticals, and Novartis. FP declares that he has received research grants and speaker's honoraria from Bayer Healthcare, Teva Pharmaceuticals, Genzyme, Merck & Co., Novartis and MedImmune. He is also a member of the steering committee for the OCTIMS study (run by Novartis). The remaining authors declare that the research was conducted in the absence of any commercial or financial relationships that could be construed as a potential conflict of interest.
